# Hepatocellular carcinomas: evolution to sorafenib resistance through hepatic leukaemia factor

**DOI:** 10.1136/gutjnl-2019-318999

**Published:** 2019-07-03

**Authors:** Orlando Musso, Naiara Beraza

**Affiliations:** 1 INSERM, Univ Rennes, INRA, Institut NuMeCan (Nutrition, Metabolisms and Cancer), Rennes, France.; 2 Gut Microbes and Health Research Programme, Quadram Institute, Norwich, UK

**Keywords:** hepatocellular carcinoma

Hepatocellular carcinoma (HCC) is the second cause of cancer-related death and it represents the leading cause of death in patients with cirrhosis.^1^ The vast majority of HCCs develop in a background of severe liver fibrosis, commonly caused by HBV or HCV infection, exposure to aflatoxin B, alcoholic and non-alcoholic steatohepatitis (NASH), as well as genetic diseases.^1^ Despite recent advances in the treatment of viral hepatitis, modelling of the dramatic rise in the incidence of NASH predicts a substantial increase in the global burden of HCC.^1^

HCC allocation to treatment options is based on tumour number, size and vascular invasion, as well as on the functional liver reserve. Although HCC aggressiveness can be inferred from these clinical parameters, screening programmes in patients at risk increasingly detect early-stage HCCs that share homogeneous clinical features, but that diverge in terms of biological and molecular features.^1^ Therefore, a more precise prediction of HCC aggressiveness is expected from a better insight on HCC heterogeneity.

Liver transplantation is the most effective curative option for HCC though it suffers from obvious limitations such as donor (organ) shortage. Alternative treatments include hepatic resection and tumour ablation, chemoembolisation and systemic therapy, which is limited to sorafenib and lenvatinib as first-line treatment, and second-line options like regorafenib among others.^1^ Sorafenib, a multikinase inhibitor with antiproliferative and antiangiogenic properties, is the gold-standard systemic treatment option improving patient survival.^1^ Still, some tumours are resistant to sorafenib, underlining the urge to understand how HCC cells develop treatment resistance.

Cancer progression results from the coevolution of a heterogeneous ecosystem involving genetic diversity, epigenetic reprogramming and remodelling of the tumour microenvironment.^2^ The tumour ecosystem selects quiescent cancer stem cells whose particular energy metabolism and detoxifying properties lead to therapeutic resistance.^2^ Cancer progression toward an increasingly aggressive disease correlates with de novo expression of oncofetal proteins and with poor prognosis.^3^ A number of oncofetal proteins have been described, including the pluripotency factors OCT4 and SOX2, as well as AFP, SKA-1, SALL4, IGF2BP, 5T4, ROR1, FOXM1, Nodal and CR1,^3^ that endow cancer cells with stemness features, such as metabolic switch, drug detoxification, quiescence, immune escape and cell plasticity (figure 1). Pluripotency confers a high-degree biodiversity to the tumour ecosystem and therefore the ability for selection of the fittest cancer cells in terms of therapeutic resistance.^2^

**Figure 1:**
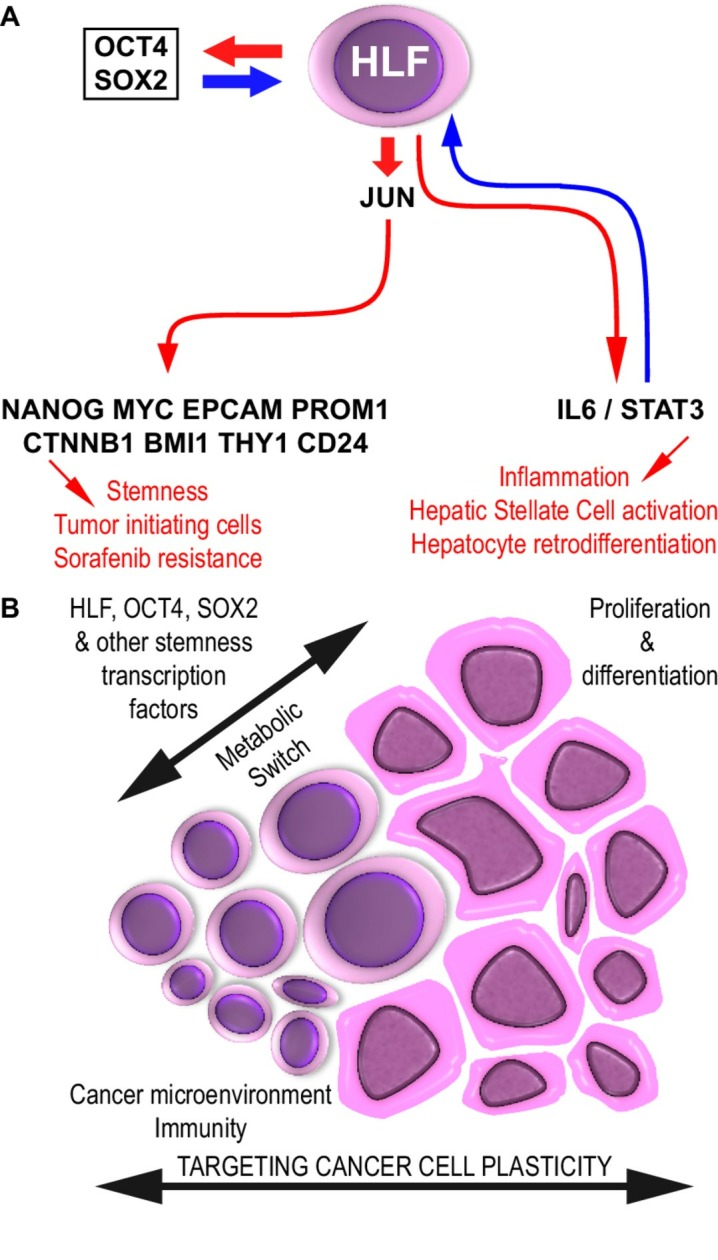
Hepatic leukaemia factor (HLF) as a paradigm for cancer cell plasticity and cancer evolution. (A) *De novo* expression of the oncofetal protein HLF is induced by the pluripotency factors OCT4 and SOX2. HLF, in turn, upregulates OCT4 and SOX2 in a positive feedback loop leading to JUN expression, AP1 signaling and expression of stem cell markers, which concur to induce tumor initiation, progression and sorafenib resistance. HLF also upregulates IL6/STAT3 in a feedforward regulatory circuit. (B) Strategies to tackle tumor evolution to cancer stem cells resistant to therapies may involve combined targeting of the metabolic/detoxification switches in cancer stem cells plus controlling cancer cell proliferation/differentiation and tumor immunity. *Left,* quiescent, metabolically fit cancer stem cells; right, proliferating and moderately-to-poorly differentiated cancer cells. Red arrows, HLF-induced effects; blue arrows, feedforward regulatory circuits. HLF, hepatic leukaemia factor.

In *Gut*, Xiang and colleagues identified hepatic leukaemia factor (HLF) as a novel oncofetal protein driving HCC progression and resistance to sorafenib.^4^ HLF is a member of the proline and acidic amino acid-rich basic leucine zipper family of transcription factors.^5^ It was first identified as part of the t(17:19)(E2A-HLF) translocation, in a rare subtype of child acute lymphoblastic leukaemia.^6^ HLF is essential for haematopoietic stem cell development and xenobiotic detoxification,^5^ and maintains quiescence of haematopoietic stem cells, protecting them from radiation or drug-induced injury and increasing drug resistance in acute lymphoblastic leukaemia.^6^

In fibrotic livers, HLF is expressed by hepatic stellate cells, which leads to a positive feedback loop further promoting HLF expression via activation of the proinflammatory IL6/STAT3 pathway.^7^ Interleukin 6 signalling plays a major role in hepatocyte retrodifferentiation and in the proinflammatory and preneoplastic microenvironment that contributes to the emergence of HCCs in severely fibrotic livers.^1 8^ Therefore, it may be hypothesised that HLF fosters the emergence of quiescent cancer stem cell populations from the onset of liver carcinogenesis. This assumption may be substantiated by evidence provided by Xiang *et al* in this issue of *Gut*,^4^ whereby the pluripotency factors SOX2 and OCT4 synergistically upregulate HLF expression and, conversely, HLF upregulates expression of the pluripotency factors SOX2 and OCT4 in a positive feedback loop. As well, HLF may upregulate stemness through NANOG, MYC and CTNNB1 (figure 1), as shown by the authors in this issue of *Gut*.^4^

The study by Xiang and colleagues also demonstrates that the induction of HLF expression in healthy adult hepatocytes leads to the expression of tumour-initiating cell (TIC)-associated markers and that the depletion of HLF protects the liver from HCC development in mice. Further mechanistic evidence established HLF as an upstream regulator of the transcription of *JUN*, a well-known oncogene^9^ that mediates the promotion of HLF-dependent TIC-like properties and tumour progression. Importantly, the authors show that the activation of the HLF/c-Jun axis confers tumour cell resistance to sorafenib, supporting previous work showing that *JUN* expression associates with sorafenib resistance *in vitro* and in patients with HCC^10^ and establishing HLF as an upstream regulator of sorafenib resistance induced by c-Jun.^4^

Overall, these results support the value of HLF expression as a prognosis marker of survival of patients with HCC by predicting resistance to sorafenib, providing a key tool for patient stratification. Still, establishing HLF as a cancer marker has obvious limitations. These include the fact that the detection of HLF must be done in tissue and that biopsies are rarely taken for diagnosis or surveillance, which is commonly carried out combining imaging with analysis of serum markers.^1^

Xiang *et al* 4 propose HLF as a therapeutic target for HCC. Based on stemness and multipotency, oncofetal proteins are attractive therapeutic targets for pharmacological approaches to treat HCC. Yet, we are still far from the ‘magic bullet’ to treat HCC: a complex multifactorial disease, involving stemness, proliferation, angiogenesis, immune exhaustion and profound metabolic changes in the tumour and its microenvironment.

From the perspective of Darwinian evolution, tumour progression results from the competition of diverse cancer cell clones for the cancer cell niche. Aggressive anticancer therapies sweep away proliferating cells, thereby releasing the competitive pressure from quiescent cancer stem cells endowed with detoxification, survival and metabolic fitness.^11^ The work by Xiang *et al* suggests that anticancer therapy should combine conventional antiproliferative and tumour mass-reducing approaches (including biotherapy and immunotherapy), with strategies to prevent the development of cancer stem cell contingents by attacking their specific attributes, such as metabolic reprogramming^8^ or the development of pluripotency features (figure 1B).^2^ Therefore, future cancer treatments may consider a wider range of cancer cell diversity to prevent the emergence of fitter cancer cell populations.

## References

[1] Forner A, Reig M, Bruix J Hepatocellular carcinoma. Lancet 2018;391:1301–14. 10.1016/S0140-6736(18)30010-2 29307467

[2] Kreso A, Dick JE Evolution of the cancer stem cell model. Cell Stem Cell 2014;14:275–91. 10.1016/j.stem.2014.02.006 24607403

[3] Ahrlund-Richter L, Hendrix MJ Oncofetal signaling as a target for cancer therapy. Semin Cancer Biol 2014;29:1–2. 10.1016/j.semcancer.2014.08.001 25111377

[4] Xiang DM, Sun W, Zhou T, et al Oncofetal HLF transactivates c-Jun to promote hepatocellular carcinoma development and sorafenib resistance. Gut 2019;68:1858–71. 10.1136/gutjnl-2018-317440 31118247

[5] Komorowska K, Doyle A, Wahlestedt M, et al Hepatic leukemia factor maintains quiescence of hematopoietic stem cells and protects the stem cell pool during regeneration. Cell Rep 2017;21:3514–23. 10.1016/j.celrep.2017.11.084 29262330

[6] Fischer U, Forster M, Rinaldi A, et al Genomics and drug profiling of fatal TCF3-HLF-positive acute lymphoblastic leukemia identifies recurrent mutation patterns and therapeutic options. Nat Genet 2015;47:1020–9. 10.1038/ng.3362 26214592 PMC4603357

[7] Xiang DM, Sun W, Ning BF, et al The HLF/IL-6/STAT3 feedforward circuit drives hepatic stellate cell activation to promote liver fibrosis. Gut 2018;67:1704–15. 10.1136/gutjnl-2016-313392 28754776

[8] Fekir K, Dubois-Pot-Schneider H, Désert R, et al Retrodifferentiation of human tumor hepatocytes to stem cells leads to metabolic reprogramming and chemoresistance. Cancer Res 2019;79:1869–83. 10.1158/0008-5472.CAN-18-2110 30837223

[9] Maeda S, Karin M Oncogene at last--c-Jun promotes liver cancer in mice. Cancer Cell 2003;3:102–4. 10.1016/S1535-6108(03)00025-4 12620404

[10] Chen W, Xiao W, Zhang K, et al Activation of c-Jun predicts a poor response to sorafenib in hepatocellular carcinoma: Preliminary Clinical Evidence. Sci Rep 2016;6:22976 10.1038/srep22976 26964667 PMC4786823

[11] Thomas F, Donnadieu E, Charriere GM, et al Is adaptive therapy natural? PLoS Biol 2018;16:e2007066 10.1371/journal.pbio.2007066 30278037 PMC6168119

